# Influence of Video Speeds on Visual Behavior and Decision-Making of Amateur Assistant Referees Judging Offside Events

**DOI:** 10.3389/fpsyg.2020.579847

**Published:** 2020-10-02

**Authors:** Vicente Luis Del Campo, Jesús Morenas Martín

**Affiliations:** Laboratory of Motor Control and Learning, Faculty of Sport Sciences, University of Extremadura, Badajoz, Spain

**Keywords:** gaze pattern, decision accuracy, video speed, offside, football

## Abstract

The aim of the study was to assess the effects of manipulating video speeds on visual behavior and decision accuracy of 10 amateur football assistant referees (ARs) when perceived video sequences of 24 possible offside actions on a large screen. An eye tracker was used to analyze participants’ visual behaviors. Signal detection analysis provided further detail of participants’ decision-level accuracy. Participants were required to decide when they perceived a player to be offside during observed sequences with different video speed manipulations (*Normal speed*, *2 speed*, and *3 speed*). Results revealed that the manipulation of video speed did not attune emergent gaze patterns differently because participants displayed similar visual behaviors, regardless of speed. However, the *normal speed* resulted in a higher percentage of correct decisions than the *3 speed*. Participants tended toward non-flagging decision bias errors when judging offsides with the *3 speed* because they made more misses, than false alarms.

## Introduction

Sport officials must interpret and correctly enforce the rules of each sport to maintain fairness and players’ safety, but also to achieve high performance in judging and making decisions about ambiguous performance situations (Bar-Eli et al., 2011). Expert judgments in sports need effective perceptual strategies to achieve improvements in the process of decision-making and anticipation (Williams and Ward, 2007). Similarly, skilled decision-making is related to the perception of relevant cues from the environment and the selection of an appropriate response (Baker et al., 2003). Previous research has demonstrated that gaze behaviors can be used as a process tracing measure to provide insights on decision-making (Hancock and Ste-Marie, 2013).

In football, Williams (2000) argued that the players displayed different perceptual strategies in 11 × 11, 2 × 2, and 3 × 3 situations because of the task nature constrained the visual strategy used. Similarly, Vaeyens et al. (2007) concluded that the number of players playing the reduced game situation influenced the visual behavior and decisions. They also showed that the experts made better decisions and a visual search strategy more adapted to the task constraints than the novels.

In judging offside situations in association football, for example, Catteeuw et al. (2009b) concluded that international assistant referees (ARs) were more accurate in detecting offside decisions and displayed longer fixations on video-projections of match play, than national-level referees. Similarly, Catteeuw et al. (2009a) found that the higher level referees made longer fixations on the offside line, and fewer flag-lag errors (i.e., to raise the flag when a player is not really offside) than national ones because they had learned to compensate for the perceptual illusion of flash-lag effect (FLE) (Gilis et al., 2009). This perceptual illusion appears when the flashed (stationary) object is perceived behind the moving target (Nijhawan, 1994). Specifically, the last defender appears to be as spatially behind the attacker receiving the ball, resulting in more cases of false alarms than misses. For a better understanding of the compensation of the FLE, some measures of verbal reports or gaze behaviors studies should be addressed in future studies (Put et al., 2013).

To facilitate acquisition of perceptual-cognitive expertise, many years of specific goal-oriented practice activities and instructions are necessary (Williams and Ericsson, 2005). In this vein, extensive experience enhances the performance of professional referees (MacMahon et al., 2007) and more hours of practice in officiating have been shown to increase the accuracy of their judgments (Pizzera and Raab, 2012). For instance, an estimation of ARs’ errors in judging offside situations is 25% (Helsen et al., 2006). To reduce the level of these error rates, specific perceptual training programs using different tools (e.g., the video and/or feedback) have been investigated to enhance assistant referees’ decision-making in the perception of offside events in football (Catteeuw et al., 2010a,c; Put et al., 2013).

A promising new paradigm to enhance the decision-making in sport is the use of “above real-time training” (ARTT). This research paradigm consists of using speeded-up video images as a realistic method to improve decision-making in elite athletes (Lorains et al., 2013a), providing greater task fidelity and representative design than computer animations (Catteeuw et al., 2010a; Put et al., 2016a). In football, studies by Lorains and colleagues have investigated the specific effects of video speed manipulations on decision-making of expert football players. For example, Lorains et al. (2013a) demonstrated that expert footballers achieved better accuracy in making decisions than sub-elite and novice groups in this off-field test with different video speed manipulations (1.25, 1.5, 1.75, and 2.0 times normal speed). The authors concluded that experts showed better performance in ARRT situations supported by faster processing and automaticity. Interestingly, the elite and sub-elite footballers reported 1.25 and 1.5 speeds as most “game-like.” Similarly, Lorains et al. (2013b) showed that expert footballers obtained better decision accuracy in a video-based choice task using ARRT than with a normal speed during the training and transfer tests.

Similarly, Farahani et al. (2017) found that training based on ARRT improved the accuracy and response times in elite footballers’ decision-making. However, these effects were limited in time because they did not last more than 2 weeks after the end of the training period. Lorains et al. (2014) also found some effects of the ARTT on visual patterns of expert football players. Specifically, the group performing ARTT made longer visual fixations on the best option to take during performance, than the normal speed and control groups after the retention tests of an intervention, where video speed was manipulated.

In football refereeing, Put et al. (2016a) reported that normal and faster speeds enhanced decisions of international assistant referees in judging offside situations. These authors concluded that training interventions for expert officials should decrease the video speed to improve response accuracy rather than increasing or varying the video speed manipulations. More recently, Spitz et al. (2018) concluded that elite referees judged ambiguous foul-play situations more severely in slow-motion replays than in real-time.

In general, these previous findings seem to reveal that the ARTT could be an effective strategy to train decision-making in football refereeing, specifically for referees at higher skilled levels. Regardless, little evidence exists about the impact of video speed manipulations in enhancing the performance of novice athletes and sport officials. In an exception, Lorains and MacMahon (2009) addressed the impact of two video speed manipulations (normal and 1.5 speed) on decision-making of footballers varying in skill level (elite, sub-elite, and novice participants). Results revealed that the high-skill group augmented differences in performance, compared to low-skill groups, as video speed was increased. They proposed that the skilled athletes seemed to need less time to process the information for decision-making because of their higher expertise levels. In this vein, Gilis et al. (2009) found that non-expert ARs achieved a lower level of performance judging offside situations when computer animations were observed at a faster speed compared to a slower speed.

However, the expert ARs learned to adopt more conservative response criteria when judged offside sequences (i.e., “not raise the flag in case of doubt”; see Put et al., 2013). Interestingly, this biased response has not been observed in studies of less experienced national ARs. For example, Luis et al. (2018) concluded that amateur ARs, but not football players, compensated for the FLE due to their embodied specific refereeing experiences. This finding could have practical implications for testing and training of football officials at different levels of experience, highlighting the need for differentiated training programs for ARs of different skill levels (Put et al., 2016a). Consequently, the research issue examined in this study concerned whether the manipulation (i.e., increasing) of video speed would reveal a compensation for the FLE in less experienced ARs when judging filmed offside events.

There is also no evidence about the contribution of manipulating video speeds in offside decisions while assessing visual behaviors of amateur ARs. Therefore, the overarching objective of this study was to address whether fast video speed manipulations (two times normal speed and three times normal speed), compared to a normal video speed condition, would influence the visual behaviors of amateur ARs and their decision accuracy in judging offside situations, perceived from a specific AR perspective. We decided to investigate increasing speed conditions due to the lack of studies testing their effects on performance of referees of lower-skill levels. Specifically, we selected the three times normal speed for the first time in studies of video speed manipulation to address whether this high video speed condition would be accompanied by decreases in amateur ARs’ decision-making performance, compared to other slower video speeds manipulated in this study.

Based on previous research, we hypothesized that perception of offside events at a normal speed would increase the efficiency of visual behaviors of these amateur ARs (e.g., performing a longer fixation on the last defender during the perception of the offsides; see Catteeuw et al., 2009a), compared to the other increasing speed manipulations. Additionally, the perception of real-time condition might lead to the emergence of better decisions in judging offside events, compared to increasing video speed manipulations because the amateur ARs might benefit from more time to process relevant stimuli from this complex situation in football (Gilis et al., 2009; Lorains and MacMahon, 2009). In accordance with this assumption, the normal video speed was expected to lead to more correct decisions and fewer incorrect decisions compared to the increasing speed manipulations, allowing compensation for the FLE.

## Materials and Methods

### Participants

A total of 10 male assistant referees from the Spanish Football Association took part in the study (*M*_age_ = 28.7; *SD* = 5.9). All participants had accumulated more than 10 years performing as ARs at the Third National Football League and/or the First Regional League. These leagues are the fourth and fifth levels of competition in the Spanish male football, respectively. Therefore, the ARs were defined as amateurs because although they had experience in a national league, and only officiated at regional-level competitions without professional experience (Swann et al., 2015). They neither reported no vision impairments, nor prior participation in talent-development programs for football officials to improve their decision-making skills in refereeing.

### Ethics

Participants voluntarily took part in the study and written informed consent, to a procedure that conformed to the Declaration of Helsinki, was obtained from the individuals for the publication of any potentially identifiable images or data included in this article. The study involving human participants were reviewed and approved by The Bioethics and Biosecurity Committee of Extremadura University (approval number 33/2018). Participants received general information about the research contexts, but were naïve to the specific objectives and hypotheses.

### Apparatus

#### Visual Behavior Assessment

An Applied Sciences Laboratories Eye Tracking ASL SE5000 recorded the visual fixations made by the participants as they observed the video speed manipulations. This device is a head-mounted, monocular eye-tracking system using corneal reflection to measure eye-line-of-gaze with respect to the field of view with an accuracy and precision of ±0.5 visual angle. The assistant referees’ gaze data were stored on a digital recording device (Panasonic NV-HS1000ECP).

#### Video Test

A total of 240 offside events were recorded from games during several training sessions of experienced football players from the Third Division of the Spanish National Football League. These events included judgments to be made whether a player had moved offside or not during the games performed in the training sessions.

The offside judgments were recorded with a digital camera (Sony DCR-SR30), and the location on the field was chosen to simulate the viewing perspective of an assistant referee (i.e., in line with the last defender, in accordance with the Rule 11 of the International Football Federation, FIFA). Specifically, the camera was placed 25 m from the goal line, 1.20 m beyond the sideline, and 1.70 m above ground level. The Kinovea software (version 8.27) was used to edit the play sequence.

The design used for the video test was based in previous studies to ensure a realistic offside situation for analysis of ARs’ perceptual and decision-making behaviors. To exemplify: (i) the interactions between the attacker receiving the ball and the last defender took place in front of the camera to simulate the correct position of the AR during the perception of these events. This methodological decision eliminated possible optical error effects as the camera angle for viewing offside events occurred at the exact place that the assistant referee should be located to observe play on the field (i.e., to eliminate an incorrect angle of view; see Catteeuw et al., 2010b); (ii) the offsides involved interactions of a small sub-group of attackers and defenders (e.g., four vs. four players visible at all times; see Catteeuw et al., 2009a); (iii) the projected game sequence contained trials with variations in viewing features, including not only small and big viewing angles but also near-medium-far distance values (Luis et al., 2015); and (iv) the sequences included distinct levels of trial difficulty, according to the spatial position of the attacker receiving the ball and the last defender (Put et al., 2014).

### Variables

The independent variable was the video speed manipulation (Level 1: real-time speed or *normal speed*, Level 2: two times normal speed or *2 speed*, and Level 3: three times normal speed or *3 speed*).

The dependent variables for visual behavior were the mean number and duration of visual fixations made by the participants to different locations of the filmed sequence of play. The visual locations of interest were the ball carrier of the attacking team and last defender who defined the offside line, and fixations made on areas of no relevant interest (the attacker receiving the ball, the ball, defensive line, and offensive line). These specific locations during the perception of offside events have been previously used in other studies (e.g., see Catteeuw et al., 2010c; Luis et al., 2018).

A visual fixation was coded when the gaze remained within one degree of visual angle of a location for a minimum duration of at least 100 ms (Piras and Vickers, 2011). Since the video speed manipulation elicited speed differing in temporal durations, the number and durations of fixations in each trial were normalized relative to the mean duration of all trials presenting offside events. The percentage viewing time for these offside locations was also calculated for dividing the time of visual fixation on each visual location by the total fixation time (of all visual locations) in each trial.

The dependent variable for decision-making performance was the response accuracy. Participants made correct decisions judging an offside action if they raised a flag when they perceived an offside situation to have occurred (i.e., termed a *hit*) or when they did not raise the flag if an attacking player was not offside (i.e., termed a *correct rejection*). In contrast, participants made incorrect decisions when they raised the flag when a player was not offside (i.e., termed a *false alarm*) or they did not raise the flag when offside occurred (i.e., termed a *miss*).

### Procedure

A selection of high quality sequences (*n* = 24), containing offside judgment decisions was video-projected onto a large screen (5 × 3 m; Hitachi CP-S310W), from the 240 initial offside events recorded during training sessions of football players. The ratio of onside and offside situations in the randomized video sequences displayed was exactly 50%. The same rate of onside vs. offside decisions was used to minimize the potential influence of any pre-conceived ideas that the ARs might have had about the likelihood of an offside taking place.

Each AR observed the same sequence of offside events at each of the three video speed manipulations. The speeds selected (normal speed and two times normal speed) were based on previous studies (Lorains et al., 2013a), and the three times normal speed was novelty used to test whether this high video speed manipulation would hinder or not AR’s decision-making performance, compared to the other speed conditions. Their order of appearance in the sequence was randomized being the same for all participants.

**Figure 1:**
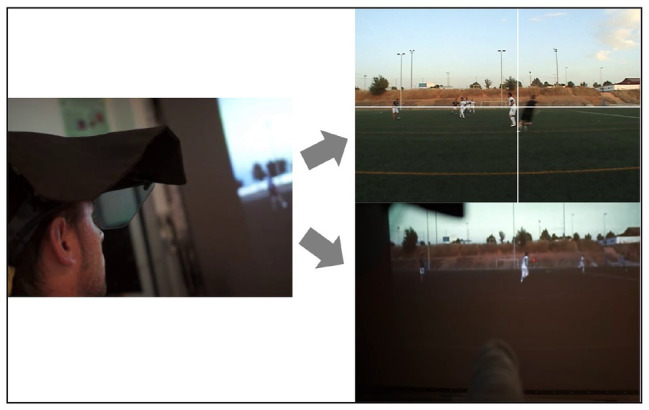
An assistant referee observing and judging a video-projected offside event in laboratory.

The procedure used in this study was identical to that undertaken by Luis et al. (2018) investigating potential differences in visual search activity of ARs and players in football. For example, the distance of the ARs to the video-projection screen was 4 m, and the image size of the football players used in the sequences was calculated to provide a realistic view of the offside judgments. Participants were required to raise a flag and press a laser pointer only in those trials that they judged to include a valid offside decision while they observed the sequence wearing the eye tracker. The laser pointer was visible on the recorded film to analyze decision accuracy. This procedure ensured that decision-making information, together with the gaze location data, was recorded to a digital device for the total number of trials. These recordings allowed a further analysis of ARs’ visual behavior and decision-making performance (see Figure 1).

The mean duration of the video sequences of offside events was about 7 s, lasting between 4 and 12 s depending on the video speed manipulation. For example, if a normal speed clip was 12 s in duration, then, the edited version of 3x that speed, was completed in 4 s. If it was edited to 2x the normal speed, then the film sequence was completed in 6 s. These temporal interval values were similar to those used by Spitz et al. (2018) in judgments of foul-play situations (e.g., 3.08 s in real-time and 12.32 s in slow-motion).

The sequence contained a pause of 3 s between trials to avoid psychological fatigue in the participants give allow them to prepare to judge the next offside event. Before the observations of the offside events, ARs undertook two practice trials to familiarize them with the experimental procedures. No performance feedback was given during the test. ARs taking part in this study had never previously viewed the offside sequences observed during the test.

### Statistical Analysis

Shapiro-Wilks and Levene analyses confirmed that the data of the dependent variables did not display a normal distribution, and then, nonparametric tests were used in this study. For the point-of-gaze data, descriptive statistics of means and SDs were used to explore the visual patterns of the participants with respect to number of visual fixations made, fixation duration times, and percentage viewing time spent on specific visual locations. A Kruskal-Wallis test was performed to determine differences between groups of video speed manipulation. The Rho Spearman correlation coefficient was calculated to address relations between visual behavior and decision-making in each video speed condition.

For the response accuracy, a Chi-Squared test was used to determine differences between video speeds in the percentages of *hits*, *correct rejections*, *false alarms*, and *misses*, and between correct (*hits* and *correct rejections*) and incorrect decisions (*false alarms* and *misses*). Additionally, The Kruskall-Wallis test was performed to determinate group differences in percentages of correct (*hits* vs. *correct rejections*) and incorrect (*false alarms* vs. *misses*) decisions made by the assistant referees. This last analysis of incorrect decisions provided information whether the groups of video speed compensated for the FLE. The Mann-Whitney test was used to determinate pairwise comparisons in these types of decisions.

The effect sizes (*ESs*), based on the correlation coefficient (*r*), were calculated to provide a better interpretation of the results. The value of the *z* distribution, obtained from performing the Mann-Whitney tests, was used to estimate the magnitude of *ES*. This statistic was reported for those non-parametric tests with significant differences between pairs of video speed conditions. Specifically, three categories of Cohen (1988) were used to interpret *ES* (small: *r* = 0.10 or *d* = 0.20, medium: *r* = 0.24 or *d* = 0.50, and large: *r* = 0.37 or *d* = 0.80). The CIs for *ES*s were calculated to provide a practical value of the study in real-world terms (Thompson, 2002). The Pearson’s *r* was converted into Cohen’s *d ES* to provide *CI* with the formula: 95% *CI* = *ES* − 1.96*se* to *ES* + 1.96*se* (Cumming, 2012). Finally, the statistical power was calculated with the G*Power software 3.1.9.2 (Faul et al., 2007) to test whether the statistically significant findings reflected true effects. A value of ≥80% power was set for analyses because it is an acceptable level to correctly reject the null hypothesis (Cohen, 1988).

A signal detection analysis (Macmillan and Creelman, 2005) was also used to analyze the response accuracy of each group in further detail. Specifically, we used d', as a sensitive index describing the assistants’ ability to discriminate between “offside” and “not offside” for the three groups of video speeds above the statistical level of chance. When d' was zero, participants were not able to discriminate above the chance level between *hits* and *false alarms* in our study. If d' differed significantly from zero, the participants were able to make this distinction. Response bias or criterion *c* was also calculated to investigate the tendency of participants to make flag errors or non-flag errors. If *c* was zero, the *false alarm* and the *miss* rates were equal. When *c* was positive, the response bias indicated that the participants tended not to press the flag in our study. When *c* was negative, the response bias indicated that the participants tended to press the flag. An alpha level of <0.05 was set for all analyses. Statistical analysis was performed using the statistical package SPSS 25.0 (Statistical Package for the Social Sciences; © 2017 SPSS Inc.).

## Results

### Gaze Behavior

Table 1 shows the mean data for fixations, fixation time, and percentage viewing time on different visual locations when participants judged the offside events at different video speeds. It is important to highlight that ARs displayed an increase of fixation time and percentage viewing time on the last defender at faster video speeds.

**Table 1:** Mean and SD (M ± DT) of visual fixations (in n°fij), fixation time (in ms), and percentage viewing time (in % respect to the 100%) for the assistant referees during the three video speed manipulations.

	Normal speed	2 speed	3 speed
**Number of visual fixations**			
Ball carrier	0.23 ± 0.37	0.30 ± 0.66	0.30 ± 0.70
Last defender	1.18 ± 0.95	1.50 ± 1.29	2 ± 2.06
Not on areas of interest^a^	3.71 ± 2.04	4.22 ± 2.16	4.60 ± 2.88
**Fixation time**			
Ball carrier	343.21 ± 736.23	235.27 ± 584.78	266.44 ± 797.31
Last defender	855.45 ± 1067.66	943.97 ± 1145.85	1102.22 ± 1363.43
Not on areas of interest^a^	2610.96 ± 1598.52	2415.47 ± 1274.62	2320.76 ± 1528.06
**Percentage viewing time**			
Ball carrier	8.33 ± 17.34	6.45 ± 14.58	6.16 ± 16.20
Last defender	22.94 ± 22.40	24.87 ± 24.61	28.86 ± 30.15
Not on areas of interest^a^	68.72 ± 24.30	68.66 ± 25.41	64.97 ± 30.90

a Not on areas of interest: attacker receiving the ball, ball, defensive line, offensive line, and gap between offensive and defensive line.

Specifically, the Kruskal-Wallis analyses did not reveal statistical differences between values of any visual variables as a function of video speed manipulations. No relations were also found between visual and decision-making variables in any of the video speed manipulations.

### Decision-Making Performance

Table 2 shows the accuracy of perceptual judgments made by ARs during judgments of offside events, highlighting that the number of accurate decisions decreased with increasing video speeds. In the *3 speed*, the ARs achieved the highest number of *correct rejections* in correct decisions, compared to the other video speed manipulations. For incorrect decisions, the ARs showed the lowest percentage of *false alarms* and *misses* in the *normal speed*, compared to the speeded video conditions.

**Table 2:** Type of decisions (in percentage respect to the 100%) made by assistant referees when judged offside events in football with three video speed manipulations.

	Normal speed (%)	2 speed (%)	3 speed (%)
Hits	45.6	39.6	23.7
Correct rejection	43	42.3	51.3
False alarm	6.3	9	10.5
Misses	5.1	9	14.5

The Chi-Squared test showed no significant differences between video speed manipulations in the proportions of decisions made (*X*^2^ = 10.74; *p* = 0.09), nor in the proportion of correct and incorrect decisions (*X*^2^ = 4.85; *p* = 0.08). The Kruskal-Wallis test also showed no significant differences between video speed conditions in the percentages of decisions when participants correctly judged the offside events (*H* = 5.58; *p* = 0.06), neither when incorrectly judging the offside events (*H* = 0.47; *p* = 0.78).

The Mann-Whitney test revealed that some pairwise comparisons between the *normal speed* and *3 speed* achieved significant differences in the percentages of decisions made by participants. These differences were found in the proportions of *hits*, *correct rejections*, *false alarms*, and *misses* (*U* = 2,186; *p* < 0.01), and in the proportions of *hits* and *correct rejections* when correctly judged the offside events (*U* = 1,599; *p* < 0.05). Specifically, the *ES* was medium when the comparison included all types of decisions (*d* = 0.51; 95% *CI*: 0.19–0.83), and small for the correct decisions (*d* = 0.39; 95% *CI*: 0.04–0.74). The power of these tests was 57.12 and 56.31%, respectively.

The ARs discriminated between “offside” and “no offside” above chance level because the sensitivity index (*d'*) was significantly different from zero for all video speed manipulations, being 2.41 for *normal speed*, 1.83 for *2 speed*, and 1.26 for *3 speed*. The ARs showed no decision bias toward making *false alarms* or *misses* because the criterion *c* showed no difference from zero, being −0.03 for *normal speed*, and 0.01 for *2 speed*. However, they reported a slight bias in response accuracy toward making non-flagging errors when they perceived offsides with the *3 speed* because the criterion *c* achieved a positive value of 0.23.

## Discussion

In this study, we investigated the impact of different video speeds on the visual behavior and decision accuracy of amateur ARs while judged a video sequence of offside events in football. More specifically, we examined whether the manipulation of the video speeds in an off-field laboratory test would lead to changes in visual behavior and decision-making of amateur assistant referees.

Interestingly, the video speed manipulation had no impact on ARs’ gaze patterns because they did not modify their visual behavior despite the task constraints of the research in the form of the video speed manipulations. Consistent with these data, the hypothesis is rejected. We expected to find more gaze behavior efficiency in ARs when they observed the offside sequences at a *normal speed* compared to the speeded video conditions (i.e., to fixate the gaze longer on areas of interest: ball carrier and last defender). In contrast, participants displayed a similar gaze pattern between the different speed manipulations. Previously, Roca et al. (2013) found no differences in the visual behavior of less skilled football players when observed life-size video sequences of dynamic 11 vs. 11 situations, containing far and near task conditions, and from the central defender’s viewing perspective. Similarly, Put et al. (2016b) found that an intensive, off-field training protocol enhanced ARs’ response accuracy through the strategy of compensating for flag errors (i.e., participants learned to compensate for the flash-lag illusion), but without changes in visual perception.

We reasoned that the increase of the video speeds during the perception of offsides may have created a “juggling effect” for the collapse of ARs’ object tracking ability (Faubert and Sidebotton, 2012). According to these authors, speeds up to the player’s boundary seem to be perceived faster and more complex than they actually are. In this vein, it is possible that the speeded videos choked this ability to track multiple stimuli, masking the possibility of amateur ARs to elaborate different perceptual strategies from the observation of offsides.

Previous studies have reported higher levels of decision accuracy when assistants fixated on the offside line for longer (Catteeuw et al., 2009a,b). However, in this study, no correlations between visual behaviors and decision accuracy were found at each video speed manipulation. It is worth noting, a tendency for higher fixation times and percentage viewing times of ARs on the last defender when video speed was increased. We argue that the increase of the video speed involved the perception and judgment of offside events that were highly temporally-constrained. This temporal limitation may have generated a hard tracking of offside stimuli, driving participants to focus their gaze for longer on this visual location of interest. Therefore, the increase of the video speed conditions during the viewing of offsides ensured longer fixations on one key stimulus of these events (i.e., the last defender who defined the offside line and the offline line). In light of these findings, we suggest that the ARs’ visual behaviors could not be taken to explain any differences in response accuracy.

For the decision-making performance, some specific effects in the ARs’ response accuracy were found between video speed conditions. To exemplify, they performed different percentages of decisions or different proportions of correct decisions when they judged the offsides at the *normal speed* compared to the *3 speed*. The *CI*s for *ES*s did not include zero or a negative number, and then, there was a 95% likelihood that a true population effect was found between the lower and the upper scores. However, this finding did not exist in the real-world because the values achieved in the power analyses were lower than 0.80 (i.e., the likelihood that the finding reflected a null effect was higher than 20%). With this low statistical power, we are aware about our limitation to state that the video speed manipulation caused true effects in ARs’ response accuracy.

In light of these statistical data, our initial hypothesis that the amateur ARs would outperform their decisions with the *normal speed* compared to the speeded video conditions is not fully accomplished. We reasoned that an underlying effect would exist in response accuracy as a result of manipulating video speed conditions because ARs showed a clear tendency to make less correct decisions and more incorrect decisions when video speeds were increased. A possible explanation to this decrease in decision-making performance during the speeded video conditions was the more distorted relative motion information between players, masking the relative position between the attacker and the last defender at the moment of the pass. We argue that the increase of the video speeds could have prevented the ARs from correcting their perception of the positions of ball carrier, attacker receiving the ball, and last defender just before the last pass; constraining a precise recall of these spatial locations at the moment of the pass (Catteeuw et al., 2010b; Put et al., 2013). Specifically, the increasing video speed inhibited a precise spatial location of the co-positioning of these three players on field at the moment to the through pass was played by the ball carrier. From this viewpoint, the fastest speed could have exceeded the capacity of amateur ARs to process the relevant stimuli of the offside events because of their low level of experience and skill in officiating (Catteeuw et al., 2009a).

The findings on perceptual sensitivity (d′) revealed that the assistants discriminated between “offsides” and “not offsides” in each video speed manipulation, even in the *3 speed*, because their sensitive index (d′) differed significantly from 0. Results suggest that the ARs had the perceptual sensitivity to discriminate these ambiguous situations in football officiating because they had accumulated enough visual and motor experiences for more than 10 years whistling as assistant referees in amateur categories. These previous observations and executions of the offside events could have helped assistants to save not biased decisions in judging offsides (Cañal-Bruland et al., 2010), showing similar rate of *hits* and *false alarms*.

Intriguingly, no differences were found between video speed manipulations for the number of incorrect decisions made. However, the assistants showed a decision bias toward errors in perceiving offsides in the *3 speed* because the criterion *c* showed a positive value different from zero (i.e., they tended to make more non-flag errors than flag errors). In elite assistant referees, this decision-level for the FLE was compensated eliminating a forward memory shift induced by this spatiotemporal illusion (Catteeuw et al., 2010a,c). However, in this study, the highest speed manipulation led to a conservative flagging strategy for the compensation of the referred perceptual illusion, when the usual decision tendency in these low-skill levels of refereeing is to make more *false alarms* than *misses* (Baldo et al., 2002; Catteeuw et al., 2010b). We suggest that the *3 speed* provoked perceptively a high task difficulty in judging offsides, with images presented too quickly on the screen (e.g., the position of the attacker receiving the ball relative to the last defender, at the moment of the pass). As the assistants had more difficulties to perceive exactly that information in the fastest videos, they gave the attacker the benefit of the doubt, showing a decisional behavior biased to not raise the flag.

Taken together, these findings posited that the video speed manipulation caused some underpowered effects in the decision accuracy (at a cognitive level) but not on the visual behavior (at a perceptual level) of amateur ARs when observed offside sequences. Specifically, the speeded video manipulations did not lead to further improvements in response accuracy. Conversely, the ARs decreased significantly the percentage of correct decisions in the *3 speed* compared to the *normal speed*, and reported a FLE in this *3 speed* toward the misses.

### Strengths and Limitations

This research study had two main advantages. First, we investigated effects of video speed manipulations on visual behaviors and types of decisions of amateur ARs (e.g., the compensation for the FLE), using signal detection analysis methodology. Second, the design used for the video test of offside decisions, based on previous studies (Catteeuw et al., 2009a, 2010b; Put et al., 2014) and perceived from the AR perspective, created fidelity and representative design for analyses of visual patterns and decision-making processes.

The study could be improved by increasing the limited amount of cases analyzed, to avoid masking observations of statistically significant differences in response accuracy levels between video speeds, which in this study remained at the level of statistical trends in the data. For example, questions exist whether there would be differences between *normal speed* and *2 speed* or between *2 speed* and *3 speed* if more assistant referees were recruited to participate in the study. This small number of assistant referees investigated could explain the low power estimation observed in this study. According to the G*Power software 3.1.9.2 (Faul et al., 2007), a total sample of 21 ARs per group should be tested to achieve the threshold of 80% power, an alpha level of 0.05, and a large *ES* (*d'* = *0.8*). Similarly, it would be interesting to address whether this tendency found of fixating longer on the offline line achieves significant levels with larger samples of participants.

Another limitation was that the experienced football players involved in the recording of the offside sequences did not receive indications about what speed execution should be performed during these specific events in football. They had freedom to perform, in a natural manner, these specific situations to accomplish with the requirements of the research team. The absence of control in this variable (e.g., to execute offsides with high, medium, and low speeds) could mask a possible effect of perceptual sensitivity in judging offsides, intrinsically linked to the speed in which the offside events were performed and recorded.

### Training Perspectives

The video speed manipulation for the refereeing performance should be introduced carefully in the perceptual trainings of the referees, according to their sport skill level. To do this, the learning task designs should state clear previously what video speed thresholds are more adequate to enhance decision accuracy for expert and amateur assistant referees. This study represents another step to elucidate this question, trying to add new evidence for a better design of perceptual training programs in development of skills in assistant referees because the influence of video speed manipulations in referees’ decision-making has received sparse interest in the literature (Spitz et al., 2018).

With these data, the speeded video conditions seem not to be a reasonable strategy for the improvement of decision performance in amateur assistant referees, at least when they perceived offsides with the *3 speed*. In contrast, the use of normal speed video clips for observations of offside sequences may prevent ARs from making incorrect decisions. It is also possible that low skilled ARs should also be exposed to different emotional and contextual variables of real-game scenarios (e.g., stress, anxiety, home advantage, and external crowd pressure) to learn how to maintain their decision-making skills under pressure. Following this relationship between emotions and decision-making, the off-field training programs undertaken in controlled-laboratory settings would gain fidelity and representativeness by including cognitive and emotional constraints that are embedded in football matches.

To do this, emotional intelligence training (e.g., the emotional regulation and pre-competitive routines; see Campo et al., 2015) or virtual-reality technology could be promising strategies to achieve transfer from laboratory to on-field scenarios when judged offside events. In this line, teaching strategies in offside situations could include, as in foul-play situations, a combination of immediate feedback about the correctness of decisions (Schweizer et al., 2011) together with a scheduling of decreasing video speed sequences (Put et al., 2016a) to train amateur ARs to better deal with the FLE.

## Data Availability Statement

The original contributions presented in the study are included in the article, further inquiries can be directed to the corresponding author.

## Ethics Statement

The study involving human participants were reviewed and approved by The Bioethics and Biosecurity Committee of Extremadura University (approval number 33/2018). The participants provided their written informed consent to participate in this study. Written informed consent was obtained from the individuals for the publication of any potentially identifiable images or data included in this article.

## Author Contributions

VL and JM equally contributed to the conception and design of the study. JM performed the measurements of the experiment. VL performed the experimental data and the statistical analyses. VL and JM wrote the first draft of the manuscript. Both authors contributed to manuscript revision, and approved the final version of the manuscript and agreed with the order of presentation of the authors.

### Conflict of Interest

The authors declare that the research was conducted in the absence of any commercial or financial relationships that could be construed as a potential conflict of interest.
